# A two-stage GAN-based instrumental variable method for causal analysis of omics data

**DOI:** 10.1093/bib/bbag071

**Published:** 2026-02-23

**Authors:** Yuan Zhou, Pei Geng, Shan Zhang, Weijia Jin, Li Chen, Feifei Xiao, Zhigang Li, Qing Lu

**Affiliations:** Department of Biostatistics, University of Florida, Gainesville, FL 32611, United States; Department of Mathematics and Statistics, University of New Hampshire, Durham, NH 03824, United States; Department of Statistics and Probability, Michigan State University, East Lansing, MI 48824, United States; Department of Biostatistics, University of Florida, Gainesville, FL 32611, United States; Department of Biostatistics, University of Florida, Gainesville, FL 32611, United States; Department of Biostatistics, University of Florida, Gainesville, FL 32611, United States; Department of Biostatistics, University of Florida, Gainesville, FL 32611, United States; Department of Biostatistics, University of Florida, Gainesville, FL 32611, United States

**Keywords:** generative adversarial networks, exposure distribution, nonlinear causal effects, deep functional neural networks

## Abstract

While considerable progress has been made in identifying candidate genes associated with complex diseases, their potential causal roles in disease etiology remain unknown. Mendelian randomization (MR) utilizes genetic variants as instrumental variables (IVs) to estimate the causal effects of disease-associated genes, thereby establishing putative causal associations and reducing spurious association findings due to confounding. To mitigate the potential bias due to the violation of IV conditions and nonlinear exposure–outcome relations in MR studies, we propose a two-stage deep learning framework, which is free from distribution assumptions of exposure given IVs and flexible to capture complex exposure–outcome relations. Specifically, we adapt the generative adversarial networks (GAN) to estimate the conditional distribution of gene expression given IVs in the first stage and apply deep functional neural networks to learn the causal relationships between gene expression and outcomes. Moreover, the proposed method is flexible to handle various data types, such as multiple gene expressions and multi-omics data. Through simulation studies under different distributions and model choices, our proposed GAN-based instrumental variable (GAN-IV) method demonstrates improved performance over the two-stage least squares method, pleiotropy-robust MR methods (e.g. MR-LINK), and state-of-the-art deep-learning-based methods (e.g. DeLIVR). A real data application on the ROSMAP dataset further illustrates that GAN-IV is capable of capturing the exposure distribution and complex nonlinear causal effect between gene expression and disease phenotype. Overall, the proposed GAN-IV framework provides a powerful and distribution-free tool for complex omics data, and accounts for unobserved pleiotropy and linkage disequilibrium.

## Introduction

Mendelian randomization (MR) studies provide important insights into the causal relationship between genes and the disease phenotype of interest, using genetic variants as instrumental variables (IVs). The IV analysis is a classic method to estimate and test the causal effect in the presence of an unobserved confounder. To ensure an unbiased estimation, the variable that is used as IV needs to satisfy at least three conditions, referred to as IV conditions [1]. If all IV conditions are satisfied, the two-stage least squares (TSLS) estimation using individual-level data or summary data is a commonly adopted MR framework. Specifically, in the first stage, the gene expression exposure is regressed on the IV to obtain the predicted gene expression, which is uncorrelated with the unobserved confounders. Then, the outcome is regressed on the predicted exposure in the second stage. When using single-nucleotide polymorphisms (SNPs) as genetic IVs, the IV conditions are often violated due to pleiotropy and linkage disequilibrium, causing bias in parameter estimation. Extensions of the TSLS framework that are pleiotropy-robust include MR-Egger [2], MR-PRESSO [3], LDA-MR-Egger [4], and MR-LINK [5]. However, all of those methods require linearity assumptions in both the IV-exposure relations and exposure–outcome relations across different levels of exposure. In practice, it is often not the case for complex diseases, where genes are linked to disease in a complicated manner (e.g. interactions). In this paper, we propose a deep learning approach to handle the pleiotropy and nonlinear exposure–outcome relationships simultaneously.

Recently, nonlinear MR estimations have been proposed to capture the complex exposure–outcome relationship including the nonparametric IV method [6], the kernel methods and orthogonal series [7], and a semi-parametric method using fractional polynomial functions and piecewise linear functions [8]. Moreover, the state-of-the-art deep learning models, as a type of nonparametric models with powerful representative ability, are applied to the estimation of nonlinear causal effects, such as DeepIV [9] and DeLIVR [10]. DeepIV [9] is a two-stage method that applies deep neural network (DNN) in both stages. In the first stage, to estimate the conditional expectation of exposure given IV using DNN, Monte Carlo simulation is applied on the DNN estimator of the conditional distribution of exposure given IV. This could lead to unstable estimation and high computational burden. In the second stage, another DNN is trained with the estimated conditional expectation and the outcome. As pointed out in He *et al*. [10], all nonparametric models, including DeepIV, will suffer from the ill-posed inverse problem. DeLIVR, on the other hand, circumvents this issue by combining parametric estimation of the conditional expectation of exposure on IV with the nonparametric estimation of the nonlinear exposure effect. This hybrid framework not only improves the estimation accuracy and computational performance at the same time, but also provides a valid statistical inference procedure. However, with the parametric distribution assumptions, the nonlinear effect estimator is only unbiased and consistent, provided the true density distribution is from the exponential family. Hence, it is desirable to develop methods that are robust in distribution assumptions and effective in capturing complex exposure–outcome relationships.

Despite the development of TSLS MR methods in extending nonlinear causal effects, little attention has been drawn to the impact of exposure distribution assumptions given IV. Unlike the linear causal effects, where the exogeneity moment condition breaks down to the conditional mean of exposure given IV regardless exposure distribution, the nonlinear causal effect models require careful study of the conditional distribution of exposure given IV. In this paper, we propose a two-stage generative adversarial network (GAN) based IV method that is free of distribution assumptions, robust to pleiotropy, and effective in capturing exposure–outcome relationships. In the first stage, we adopt the conditional GAN, a type of generative model, to fit the conditional distribution of exposures given IV and further obtain the fitted conditional mean exposures. GAN-based models have been proven to be a likelihood-free tool to model complex underlying data distributions that outperform other deep-generative models, especially in high-dimensional datasets [11]. Although GAN models do not provide an explicit density function estimator, a well-trained GAN model produces high-quality samples that follow the desired distribution, considering the underlying complexity. In MR studies that adopt a two-stage estimation strategy, samples from the underlying distribution are sufficient for the second stage estimation. In the second stage, with the fitted conditional mean exposure values from the first stage, we adopt the deep learning frameworks to provide nonparametric estimation of the nonlinear causal effects. These deep learning frameworks are versatile to accommodate both scalar and multiple gene expression data. Furthermore, to utilize tag-SNP data in handling pleiotropy, we adopt a multimodal functional deep learning (MFDL) model [12] in the second stage. As discussed in simulation studies in Section 3, when accounting for the pleiotropy introduced by multiple pathways, the flexible multimodal structure of MFDL can incorporate tag-SNPs, providing robust estimators that outperform competing methods.

This paper is organized as follows: in Section 2, we first introduce notations and the classic TSLS, then describe our GAN-IV framework; in Section 3, we design three simulation studies including non-Gaussian underlying exposure density, nonlinear causal effect of exposure, and confounding caused by LD effect. We compare our proposed model with other competing methods under different evaluation criteria. In Section 4, we further evaluate our models and competing methods in a real data analysis. Finally, in Section 5, we summarize our findings and discuss potential limitations of our proposed framework.

## Methods

In this section, we first present the causal inference model setting and the classic TSLS in Section 2.1 and then introduce the foundational GAN model in Section 2.2. In Section 2.3, we present our proposed two-stage GAN-based IV method for causal analysis using omics data.

### Model setting and TSLS

**Figure 1:**
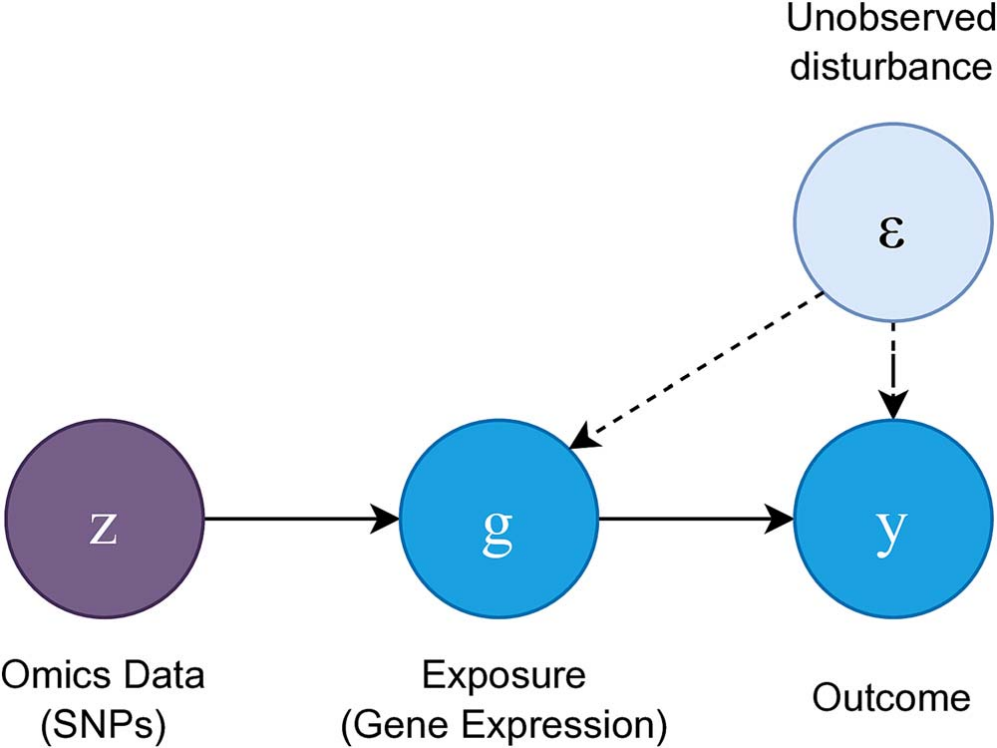
Causal diagram of an MR problem using genetic IVs.

In the causal inference framework, we are interested in the causal effect of the exposure $G$ (e.g. gene expression) on the outcome $Y$, in the presence of an unobserved disturbance $\varepsilon $, as shown in Fig. 1. Let 


(1) \begin{align*}& Y = f(G)+\varepsilon , \,\,E(\varepsilon)=0,\,\, E(\varepsilon|G)\neq 0,\end{align*}

allowing $\varepsilon $ to be correlated with exposure $G$.

Let $Z$ be the IV for exposure $G$. The exogeneity condition $E(\varepsilon |Z=z)=0$ yields 


(2) \begin{align*}& E(Y|Z=z) = E[f(G)|Z=z] +E(\varepsilon|Z=z) = \int f(g)dF(g|z),\end{align*}

where $F(g|z)$ is the conditional distribution function of G given $Z=z$. We see that the conditional distribution $F(G|Z)$ plays a critical role when linking the outcome to the IV, especially when the causal effect $f$ is nonlinear. $E(Y|Z)$ and $F(G|Z)$ are two directly estimable functions from the data, and the estimation of $f(g)$ can be achieved. In practice, estimation with the directed acyclic graph in Fig. 1 is implemented in two stages: first estimating $F(G|Z)$, and then estimating ${f}(g)$ after replacing $F$ with the estimator $\hat{F}$. In the special cases of linear effects between both $(Z, G)$ and $(G, Y)$, i.e. 


(3) \begin{align*}& G = \alpha_{0}+\alpha_{1} Z+\varepsilon_{G},\space\space Y = \beta_{0} +\beta_{1} G+\varepsilon_{Y},\end{align*}

(2) is simplified as $E(Y|Z=z) = \beta _{0} + \beta _{1}E(G|Z=z)$ and the estimation process can be described using a TSLS model. The TSLS model solves two linear regressions below to obtain an unbiased and consistent estimator $\hat{\beta _{1}}$. With the linearity and homogeneity assumptions, TSLS first obtained $\hat{G} = \hat E(G|Z=z)$ from the first-stage linear regression, then plugged in $\hat{G}$ into the second-stage linear regression and solved for $\hat{\beta _{1}}$. The closed form of the TSLS estimator is $\hat{\beta }_{1, TSLS} = \frac{{Cov}(Z,Y)}{{Cov}(Z,G)}$.

### Generative adversarial network and the conditional extension

For the two-stage methods using (2), a key component is the estimation of the conditional distribution of $F(g|z)$, which represents the exposure distribution given the IV. To accurately estimate $F(G|Z)$ in the first stage, we apply a conditional GAN model to estimate the conditional distribution. The conditional GAN consists of two models that compete with each other: a generative model denoted as $G_{\theta }$ with parameters $\theta $ that map from a noise distribution $s \thicksim p_{s}$ to a generator distribution denoted as $p_{NetG}$; a discriminative model denoted as $D_\phi $ with parameters $\phi $ that determine the inputs as real versus “fake” (i.e. generated from $p_{NetG}$). Both models are nonlinear mapping functions. In our case, we construct them using two DNN models with different model structures. The generator and discriminator are trained iteratively as follows: 1) sampling noise $s$ and feeding both the generated data $G_\theta (s)$ and observed data $g$ to the discriminator $D_\phi $; 2) calculating $D_\phi (G_\theta(s))$ and $D_\phi (g|z)$, respectively, producing a probability ranging from $[0,1]$; 3) calculating an objective function and updating parameters $\theta ,\phi $ alternately until the objective function converges. With IV information, we propose the conditional GAN by modifying the classic minimax objective function of GAN as 


(4) \begin{align*} & \min_\theta \max_\phi V(G_\theta,D_\phi)=\min_\theta \max_\phi E_{g\thicksim p_{data}} [\log D_\phi(g|z)] \nonumber\\ &\quad + E_{s \thicksim p_{s} (s)}[log(1-D_\phi(G_\theta (s|z)))] ,\end{align*}

which attains the global optimum at $p_{NetG}=p_{data}$ [13]. When this global optimum is reached, data points generated from $\hat{G}_\theta (g|z)$ is considered to have the same distribution as the observed data. Here, $Z$ can be any auxiliary information, as shown in Mirza *et al* [14]. The estimator $\hat{G}_\theta (g|z)$ serves as a generating distribution for samples needed in the second stage.

In this paper, the generator $G_\theta $ of GAN is implemented as a DNN with three hidden layers, using the tanh activation function in all layers except for the output layer. The discriminator $D_\phi $ is implemented as a DNN with three hidden layers, using ELU activation function in hidden layers and sigmoid activation function in the output layer. We train the model with non-saturating generator loss $L_{G}$ and BCE discriminator loss $L_{D}$ with $R_{1}$ gradient penalty in discriminator nets [15] defined as 


\begin{align*} L_{G} &=-E_{z\thicksim p_{z}, s\thicksim p_{s}}[-logD_\phi(G_\theta(s|z), z)],\quad L_{D} =1/2(L_{D}^{real}+L_{D}^{fake})+L_{D}^{R_{1}}, \\ L_{D}^{real} & = E_{(g|z)\thicksim p_{data}}[-logD_\phi (g|z)],\\ L_{D}^{fake}& = E_{z\thicksim p_{z}, s\thicksim p_{s}}[-log(1-D_\phi (G_\theta(s|z), z))],\\ L_{D}^{R_{1}} & = \frac{\lambda}{2} E_{(g|z)\thicksim p_{data}}[\Vert \triangledown_{g} D_\phi (g,z)\Vert_{2}^{2}]. \end{align*}

It can be shown that, for any fixed generator that induces $p_{G}(g|z)$, the discriminator that optimizes the loss function of (4) is 


\begin{align*}& D^\star_{G} (g,z) = \frac{p_{data}(g|z)}{p_{data}(g|z)+p_{G}(g|z)} \end{align*}

The proof is similar to the proof of Proposition 1 in Goodfellow *et al*.. If plugging $D^\star $ into (4), the minimax game can be reformulated as 


\begin{align*} \max_\phi V(G_\theta,D_\phi) &= E_{z\thicksim p_{z}}[E_{g\thicksim p_{data}(\cdot|z)} [\log D^\star_{G} (g|z)]\\&\quad + E_{g \thicksim p_{G} (\cdot|z)}[log(1-D^\star_{G} (g|z))]]\\ &=E_{z\thicksim p_{z}}[-log4+2\space JSD(p_{data}(\cdot|z) \Vert p_{G}(\cdot|z))]. \end{align*}

Here $JSD(p_{data}(\cdot |z) \Vert p_{G}(\cdot |z))$ is the Jensen–Shannon divergence between the true conditional distribution and the generated conditional distribution. Since $JSD(p_{data}(\cdot |z) \Vert p_{G}(\cdot |z)) \geq 0$, the optimality of the loss function is equivalent to $p_{G}(\cdot |z) = p_{data}(\cdot |z)$. In practice, to check for optimality, we look for whether the discriminator accuracy equals 0.5 and the discriminator loss is almost the same as the generator loss when convergence. When achieving optimality, we obtain a sampler $\hat G_\theta (s|z)$ for the conditional distribution $p(g|z)$. For a fixed $Z=z$ and independent noises $s_{1},..., s_{M}$, let $\tilde{g}_{m} = \hat G_\theta (z, s_{m}), m = 1,...,M$ be samples generated from the sampler $\hat G_\theta (s|z)$. Summarizing the $M$ data points, we can obtain the Monte Carlo estimator of the conditional mean for $Z=z$: 


\begin{align*}& \hat{E}(G|Z=z) = \frac{1}{M}\sum_{m}^{M} \hat G_\theta(s_{m}|z) \end{align*}

In our implementation, both DNNs are optimized with the Adam optimizer [16] and trained for a fixed epochs of 50 000. The training process is described in Algorithm 1. 



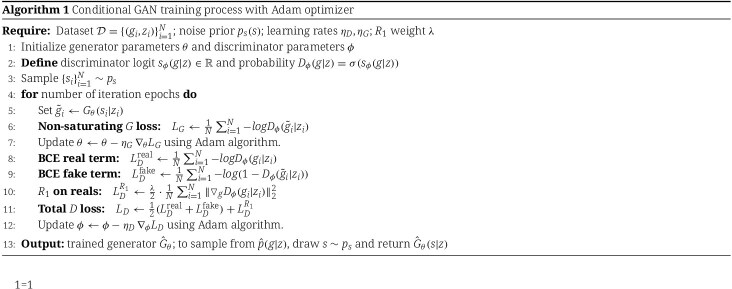



To evaluate the estimation performance of the exposure distribution given IV in the first stage, we introduce the following three criteria that will be used in the simulation study. Denote the underlying relation between exposure and IV as $h(z)$, i.e. $G = h(Z) + \varepsilon _{G}$.

(1) Bias between the estimated exposure $\hat g$ and the true mean exposure given IV $E(g|z)$  
  \begin{align*}& Bias(\hat{G},h(Z)) = \frac{1}{n} \sum_{i=1}^{n}|\hat{g_{i}}-h(z_{i})| \end{align*}
  For instance, $h(z) = \beta _{0} + \beta _{1} z$ in the classic setting of (3) and the estimated exposure using TSLS is computed as $\hat g_{TSLS} = \hat \beta _{0} + \hat \beta _{1} z$, where $\hat \beta _{0}$ and $\hat \beta _{1}$ are the TSLS estimators. The estimated exposure using GAN $\hat g_{GAN}$ is calculated as
  \begin{align*}& \hat g_{i,GAN} = \frac{1}{M}\sum_{m=1}^{M} \hat G_\theta(s_{i,m}|z_{i}),\,\, i = 1,...n \end{align*}
(2) KL divergence approximation between generated data points and data points from the true underlying distribution [17]. For the $i$th sample, let $p_{i}(\cdot )=p(g\mid z_{i})$ and $\widehat p_{i}(\cdot )$ be the true and estimated conditional density, respectively. For TSLS, $\widehat p_{i,TSLS} = \mathcal N(\hat \alpha _{0}+\hat \alpha _{1} z_{i},\hat \sigma _{G}^{2})$. For GAN, there is no explicit form of the estimated density. Instead, we draw $M$ samples from the trained generator $\hat G_\theta $, denoted as $\{\tilde g^{(m)}_{i}\}_{m=1}^{M}$, and $\widehat p_{i,GAN}$ is obtained as a Gaussian kernel density estimator (KDE) using $\{\tilde g^{(m)}_{i}\}_{m=1}^{M}$. To calculate the KL divergence, we draw $\{x^{(m)}_{i}\}_{m=1}^{M} \sim p_{i}$, and calculate
  \begin{align*}& \widehat D_{\mathrm{KL}} = \frac{1}{n}\sum_{i=1}^{n} \widehat D_{\mathrm{KL}}\big(p_{i} \,\Vert\, \widehat p_{i}\big) \\& =\; \frac{1}{n}\sum_{i=1}^{n}\frac{1}{M}\sum_{m=1}^{M} \Big\{\exp\!\big[\log{\widehat p}(x^{(m)}_{i})-\log{p}(x^{(m)}_{i})\big] \;-\; 1 \\& \quad -\; \big[\log{\widehat p}(x^{(m)}_{i})-\log{p}(x^{(m)}_{i})\big]\Big\}. \end{align*}
(3) Maximum mean discrepancy (MMD) [18] between the estimated exposure distribution and true exposure distribution. For the $i$th sample with instrument $z_{i}$, we draw $M$ samples $\{\tilde g^{(m)}_{i}\}_{m=1}^{M} \sim \widehat p(g\mid z_{i})$ from the first–stage model (GAN and TSLS), and $M$ samples $\{g^{(m)}_{i}\}_{m=1}^{M} \sim p(g\mid z_{i})$ from the ground-truth density. Let $k(\cdot ,\cdot )$ be a Gaussian RBF kernel and form a Gram matrix $K$ over stacked data set $\{\tilde g^{(m)}_{i}\}_{m=1}^{M} \cup \{g^{(m)}_{i}\}_{m=1}^{M}$. Then partition $K$ into three blocks $K_{\tilde{g}\tilde{g}}$, $K_{\tilde{g}g}$, and $K_{gg}$, each of size $M\times M$. Denote the min-max scaler transformer as $\phi (x) = (x-\min _{x})/(\max _{x}-\min _{x})$, and the estimator of squared MMD is defined as
  \begin{align*} \widehat{\mathrm{MMD}}^{\,2} &= \frac{1}{n}\sum_{i=1}^{n}\widehat{\mathrm{MMD}}^{\,2}_{i} \\& =\frac{1}{n}\sum_{i=1}^{n} \Big\{\overline{\phi(K_{\tilde g_{i}\tilde g_{i}})} \;-\; 2\,\overline{\phi(K_{\tilde g_{i} g_{i}})} \;+\; \overline{\phi(K_{g_{i}g_{i}})}\Big\}, \end{align*}
  where $\overline{A}$ is the mean of all entries of $A$. It has been proven that squared MMD is equivalent to complete agreement between two distributions [18]. The model estimator is closer to the true data-generating mechanism with lower values of $\widehat{\mathrm{MMD}}^{2}$.

### The proposed two-stage framework: GAN-IV method

After obtaining the trained sampler $\hat G_\theta (s|z)$ and the estimated conditional mean exposure from the first stage, in the second stage of causal effect estimation, we propose to use deep learning models to capture the nonlinear exposure–outcome relationship. Particularly, we consider two scenarios: multiple gene expressions over time and gene expression with tag-SNP data. To handle multiple gene expression data, we denote $g=(g_{1}, g_{2},...,g_{p})$ as the exposure data collected at different time points that can be considered as functionals. We apply the deep functional neural network (DFNN) [19] to efficiently utilize the nearby locations of genetic data, yielding robust estimation of the nonlinear causal effects $f(g)$. DFNN treats $g$ as an ordered sequence and applies basis expansion and functional linear models in functional data analysis to obtain smoothed functional curves as the input and hidden layers. The case of gene expression with tag-SNP data available is particularly useful in the presence of pleiotropy, where the bias introduced by the LD effect in eQTLs can be corrected using individual-level tag-SNP information [5]. In this case, the multimodal extension of the DFNN with the application to multi-omics data [12] is well suited to integrate both gene expression and tag-SNP data, which introduces separate basis functions built on each omics modality. Compared with concatenating all inputs into a column into a single vector, the multimodal structure achieves better latent representation and captures correlations between different data sources.

**Figure 2:**
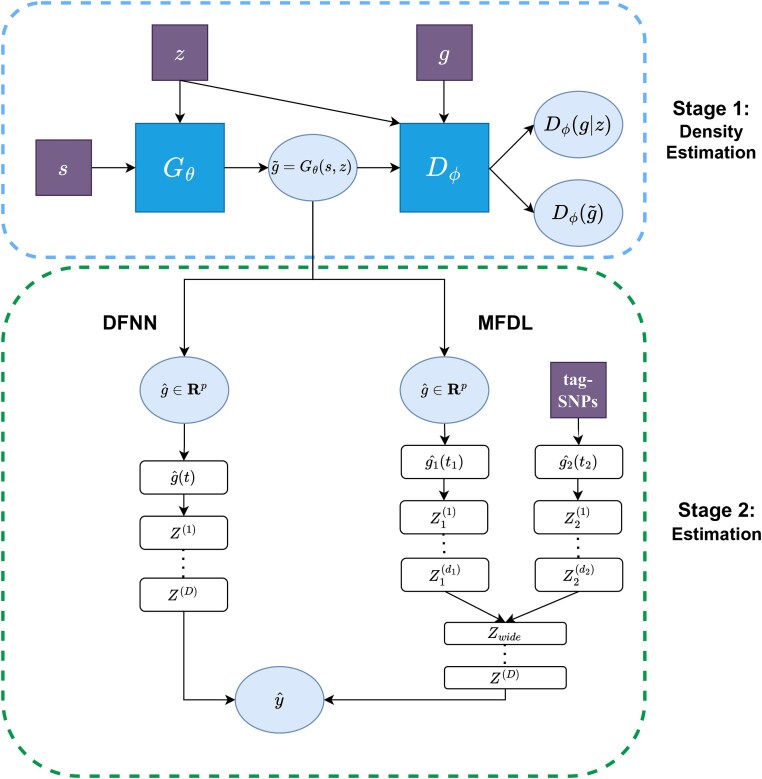
Hierarchical structure of the proposed GAN-IV framework.

In practice, we construct a DFNN model with two hidden layers and apply the basis expansion with B-Spline functions of $n_{basis}=10$ and order $3$. The activation function of DFNN is the ReLU function except for the output layer. The mean squared error (MSE) is used as loss function. The model is optimized using the Adam optimizer with $L_{2}$ regularization and early stopping. The learning rate and $L_{2}$ regularization parameter are determined via grid search using a three-fold cross validation. The detailed implementation settings are described in the appendix. In summary, the two-stage GAN-IV method uses the GAN model to estimate the conditional density in the first stage and the deep learning models to estimate causal effects using omics data in the second stage. The workflow and the architecture of the whole framework are shown in Fig. 2.

To comprehensively evaluate the causal effect modeling performance in the second stage, we introduce the following criteria: bias, MSE, and RV correlation coefficient. Let $\mathbf{Y}$ and $\hat{\mathbf{Y}}$ be the vectors of all observations and predicted values of $Y$, respectively. 


\begin{align*}& \begin{aligned} Bias(\hat{\mathbf{Y}},\mathbf{Y}_{0}) &= 1/n \sum_{i=1}^{n}|\hat y_{i}-y_{0}|, \\ MSE(\hat{\mathbf{Y}},\mathbf{Y}) &= 1/n \sum_{i=1}^{n}(\hat{y_{i}}-y_{i})^{2},\quad RV(\hat{\mathbf{Y}},\mathbf{Y}) = \frac{tr(\mathbf{Y}\mathbf{Y}^{T} \hat{\mathbf{Y}}\hat{\mathbf{Y}}^{T})}{\sqrt{tr(\mathbf{Y}\mathbf{Y}^{T})^{2}tr(\hat{\mathbf{Y}}\hat{\mathbf{Y}}^{T})^{2}}}. \end{aligned} \end{align*}

The bias criterion refers to the absolute difference between $\hat{Y}$ and the true unobserved $Y_{0}$, while MSE evaluates the modeling accuracy between $\hat Y$ and the observed $Y$. The RV correlation coefficient is a multivariate extension of the squared Pearson’s correlation.

## Simulations

In this section, we designed three simulation studies to evaluate the performance of our proposed model compared with the TSLS model, MR-LINK, and DeLIVR. Simulation 1 shows the model performance of the conditional density estimation in stage 1 under various distribution choices, and Simulation 2 presents the estimation performance in stage 2 under different nonlinear causal effects. Simulation 3 focuses on the model performance when there is pleiotropy through LD effect in the IV.

### Simulation 1: exposure distributions

This simulation evaluates the exposure distribution estimation in the first stage of the proposed GAN-IV method. To mimic real-world genetic architecture, we generated IV SNPs for 1000 samples assuming a minor allele frequency (MAF) of 0.2 under Hardy–Weinberg Equilibrium. To evaluate the robustness of our model against density assumption violations, the error terms of the exposure variable $\varepsilon _{G}$ were generated from four distributions: Normal, Laplace, Gamma, and a Mixture of Normals. Furthermore, we assume that $Y$ is a scalar, following a linear relationship with exposure $G$, an unobserved confounder term correlated with first stage noise term, and generate $Y$ as a scalar. Detailed data generation mechanisms and mathematical formulations are provided in the appendix.

In this simulation setting with linear causal effects and exposure–IV relations, DeLIVR and TSLS share the same modeling results, while MR-LINK is not applicable with no pleiotropy. Hence, only the performance of TSLS and GAN-IV is evaluated separately at the first and second stages. Since TSLS assumes normality of the exposure distribution, the TSLS distribution estimator is a normal distribution $\mathcal N(\hat \alpha _{0}+\hat \alpha _{1} z_{i},\hat \sigma _{G}^{2})$, where $\hat \alpha _{0}$, $\hat \alpha _{1}$, and $\hat \sigma _{G}^{2}$ are the linear regression estimators in the first stage as described in (3); our proposed GAN-IV method learns the exposure distribution through GAN. The three criteria defined in Section 2.2, bias, KL divergence and MMD, are used to evaluate the estimation effectiveness.

**Figure 3:**
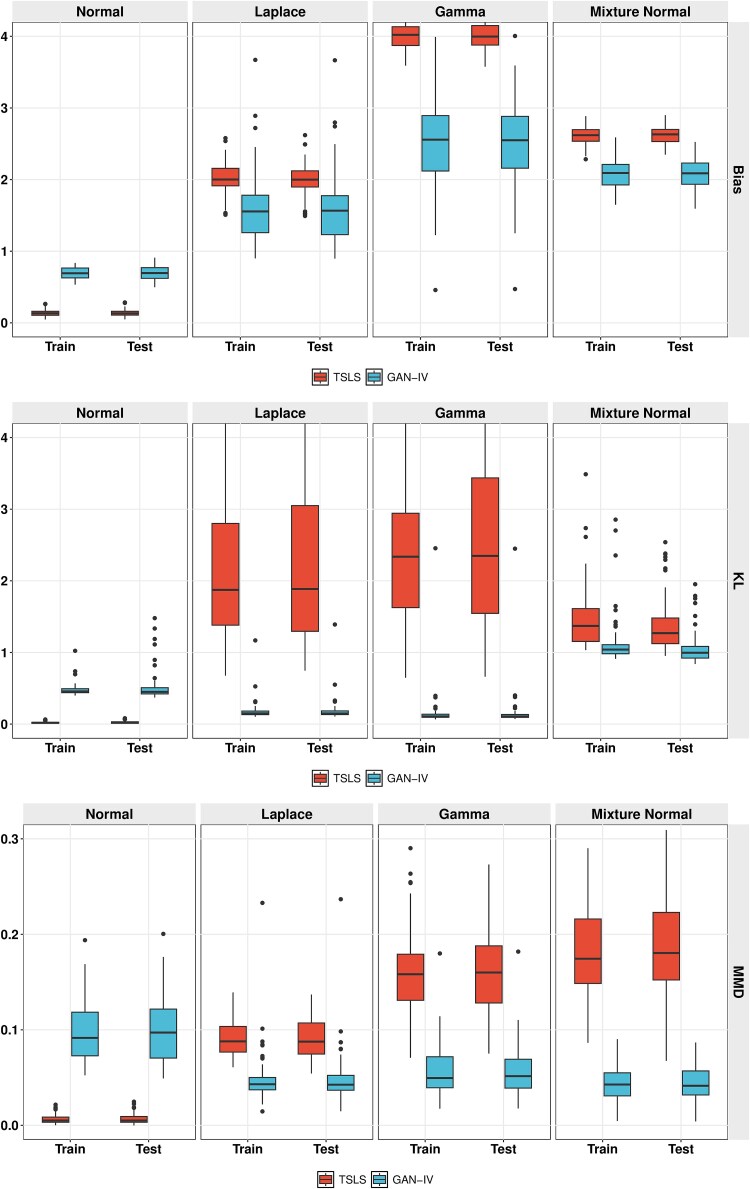
Distribution estimation performance in stage 1 regarding bias, KD divergence, and MMD for four choices of distributions.

Similar to most two-sample estimation procedures, in stage 1, we use 20% of samples to train a density estimator, then apply the estimated transformations on the rest 80% of Z dataset to prepare the estimated exposure $\hat{G}$ for the second stage model fitting. For the model fitting on the rest 80% of the dataset $G, \hat{G},Y$, we further split it into a training set and a testing set using a ratio of 4:1. For both models compared in this simulation, the stage 2 estimators are trained using training data and evaluated on testing data. The results of 100 replicates are summarized and presented in Fig. 3.

As we can see in Fig. 3, all three figure panels show that under all non-normal underlying distributions (Laplace, Gamma, and Mixture Normal), GAN-IV outperforms TSLS. Meanwhile, unsurprisingly, TSLS achieves lower but comparable performance with GAN-IV under the normal distribution. Moreover, GAN-IV attains robust performance for all four distributions and the three criteria, while TSLS shows inconsistent performance in several cases, such as high bias in Gamma and unstable KL in Laplace and Gamma.

### Simulation 2: nonlinear causal effects

In this simulation, we focus on the estimation of the nonlinear causal effect under different chosen nonlinear exposure–outcome relationships. Similar to Simulation 1, $Z$ and $G$ are generated following the same procedure with the exposure $G$ following a normal distribution. To construct the outcome variable, the phenotype $Y$ is generated by a latent function of the exposure, $f(G)$, with an unobserved confounder U and an additive noise term $\varepsilon _{Y}$. Four different nonlinear forms are chosen as the causal relation $f(g)$, including the cosine function (Cos), the polynomial function (Poly), the linear function with a two-way interaction term truncated with a threshold (rwo-way thres), and the linear function with a three-way interaction term truncated with a threshold (three-way thres). The detailed simulation settings are described in the appendix.

We compare our proposed GAN-IV model with TSLS and DeLIVR [10]. Note that MR-LINK is not applicable in this setting without pleiotropy. The data splitting procedure is the same across all three methods as in simulation 1. In this simulation, when applying DeLIVR, as the first step, a linear regression is fitted using 20% of instrument-exposure data $(Z,G)$. This linear model is then applied on the rest 80% to obtain $\hat{G}$. In the second step, a shallow neural network with one hidden layer using the early-stopping technique is trained in the subtrain-valid dataset, and finally evaluated separately on the whole training dataset and testing dataset. The hidden nodes, learning rate, and patience in early-stopping are fixed as recommended values in He *et al*. [10].

**Figure 4:**
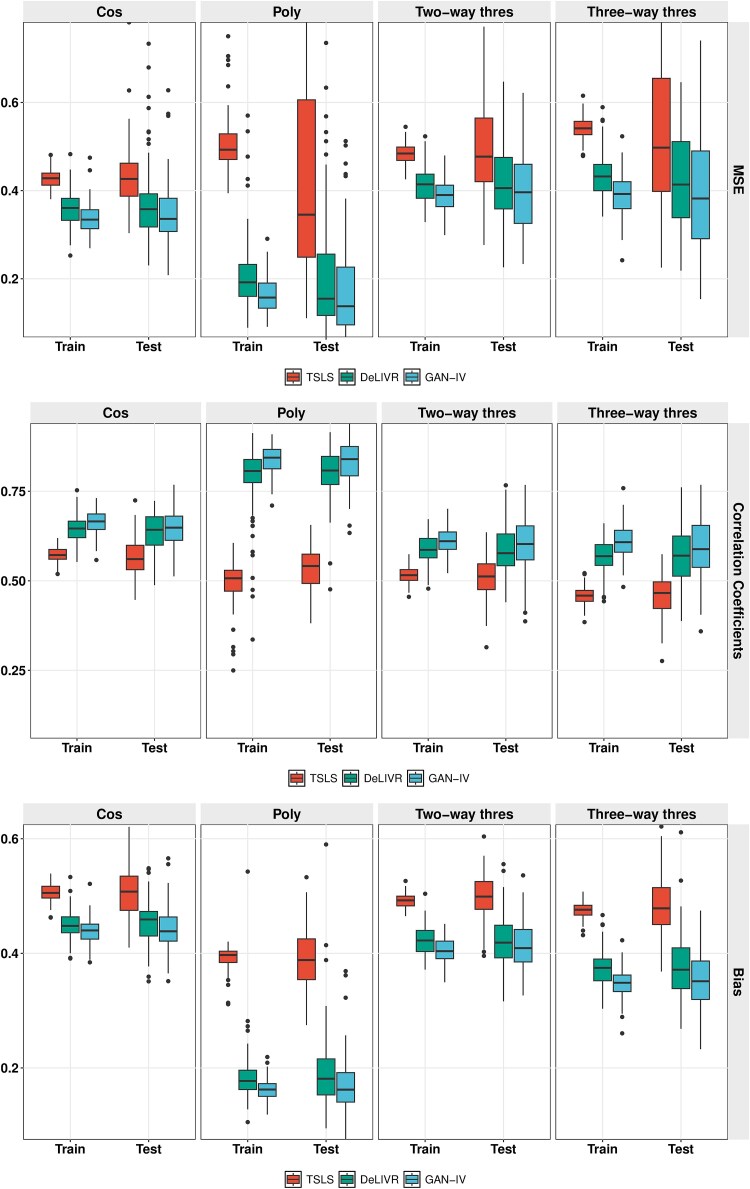
Causal effect estimation performance in stage 2 regarding MSE, RV correlation, and bias for four choices of nonlinear causal effects.

As shown in Fig. 4, GAN-IV achieves the best performance for all four nonlinear relationships and the three criteria, followed by DeLIVR and TSLS. The performance of TSLS gets worse when the causal relations deviate further from linearity. For instance, the MSE of TSLS is comparable with GAN-IV and DeLIVR in the two interaction models, while the MSE disparity becomes more evident in the Poly and Cos cases. On the other hand, the performance of GAN-IV and DeLIVR is robust across all four nonlinear causal choices, while GAN-IV consistently outperforms DeLIVR. The finding that GAN-IV and DeLIVR produce similar estimation performance in the second stage could be resulted from the similar deep learning architecture in the second stage.

### Simulation 3: causal effects with pleiotropy and LD

In genetic data analysis, the IV assumptions in causal inference can be violated in the presence of linkage disequilibrium (LD) between the eQTL variants used as IVs, or in the presence of pleiotropy. In those cases, the presence of LD brings overfitting in MR, hence extended MR methods, such as MR-LINK, are developed to correct for LD and pleiotropy. MR-LINK uses summary statistics of an exposure combined with individual-level data on the outcome to estimate the causal effect of an exposure from IVs, and to correct for pleiotropic effects using genetic variants that are in LD with these IVs.

In this simulation, we simulate a causal scenario with pleiotropy through LD where the outcome is affected through two pathways from correlated SNPs. To mimic the real data scenarios with LD effects in SNPs and fairly compare with MR-LINK, we use the same data generating procedure as in van Der Graaf *et al*. [5]. For the genotype simulation, we downloaded Chromosome 2 genotypes from the 1000 Genomes Project [20] Phase 2 and extracted the interval chr2:100 000 000–105 000 000 on GRCh37. We downloaded the HapMap genetic map for chromosome 2 and computed each variant’s genetic position by linear interpolation between two flanking map markers, where local recombination rate is the segment slope. No extrapolation was performed beyond the map range. We then simulated $1500$ samples using HAPGEN2 [21]. The full cohort was further split into three cohorts: exposure (500), reference (500), and outcome (500). This step yielded 3101 variants in the region. For the exposures, we generated two sets: the observed exposure $g_{E}$ and the unobserved exposure $g_{U}$. We first randomly selected 10 causal SNPs $Z_{E}$ from the entire genetic region for the observed exposure, then for each causal IV in set $Z_{E}$, we identified an SNP in LD and included it in set $Z_{U}$ for the unobserved exposure, making the pleiotropic proportion 100%. We simulated two exposure sets: the observed exposure $G_{E}$ and the unobserved exposure $G_{U}$. To represent the genetic architecture under LD effect, both $G_{E}$ and $G_{U}$ are generated as a linear combination of their corresponding causal SNPs $Z_{E}$ and $Z_{U}$ with an independent confounding term. Finally, the phenotype $Y$ is generated from both linear and nonlinear causal effect models. For the linear scenario, $Y$ is constructed as a linear function of $G_{E}$ and the confounder $G_{U}$; for the nonlinear scenario, the construction of $Y$ incorporates quadratic and trigonometric transformations of $G_{E}$ besides the linear term of $G_{E}$ and $G_{U}$. The specific implementation is provided in the appendix.

**Figure 5:**
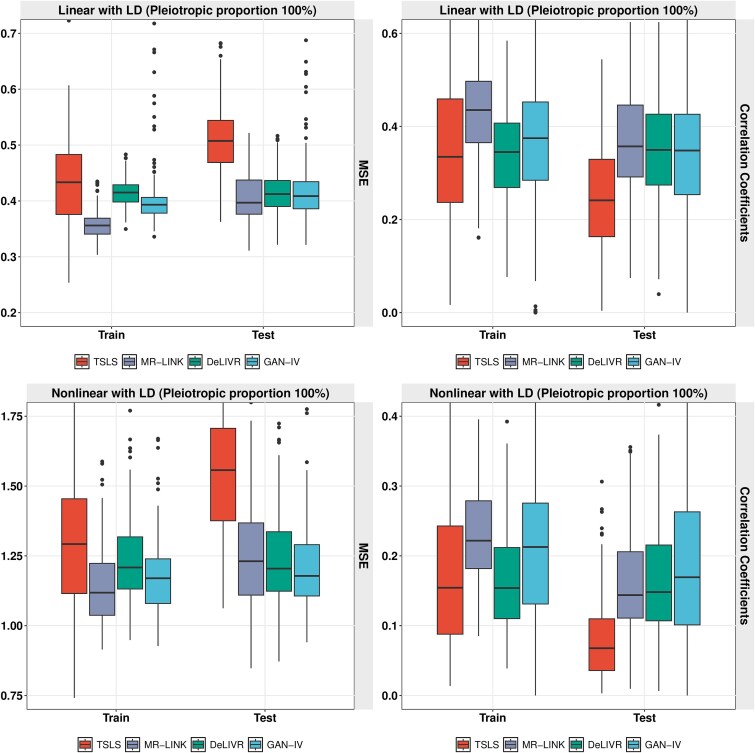
Causal effect estimation performance for linear (upper panel) and nonlinear (lower panel) causal relationships with pleiotropy proportion of 100%.

The methods compared in this simulation include TSLS, MR-LINK, DeLIVR, and our proposed model, GAN-IV. For each simulated dataset, we first applied MR-LINK to obtain qualified IV SNPs and further identified a set of tag-SNPs that are correlated with the IVs. For TSLS and GAN-IV, tag-SNPs are included in the second stage. For the proposed GAN-IV method, we apply the MFDL model in the second stage to accommodate the tag-SNPs. The results of the MSE and RV correlation for the chosen linear and nonlinear causal relations are summarized in Fig. 5. For the linear causal relation with LD effects, MR-LINK, GAN-IV, and DeLIVR share similar performance. They all outperform TSLS, although MR-LINK shows slight overfitting. For nonlinear causal relation with LD, our proposed GAN-IV achieves the best performance in the test set in terms of both MSE and RV correlation among the four methods, followed by DeLIVR and MR-LINK. Overfitting remains an issue in TSLS in the nonlinear case.

**Figure 6:**
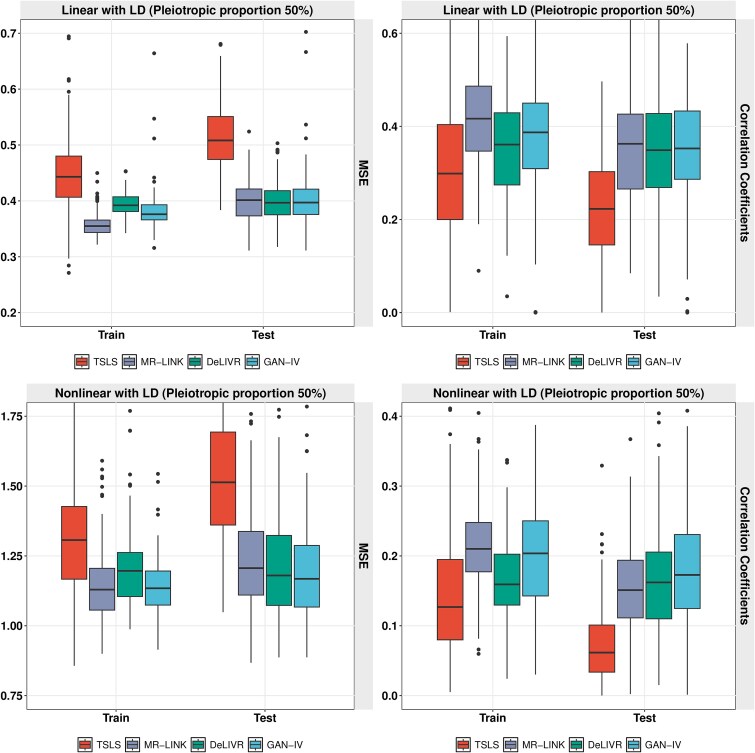
Causal effect estimation performance for linear (upper panel) and nonlinear (lower panel) causal relationships with pleiotropic proportion of 50%.

We further evaluated the effect of pleiotropic proportion by conducting an additional simulation. We consider a pleiotropic proportion of 50%, i.e. for 10 causal IVs in set $Z_{E}$, we randomly selected five of these IVs and included their corresponding in-LD SNPs in the set $Z_{U}$. The data generating process was the same as described above. Figure 6 shows results summarizing MSE and RV correlation for linear and nonlinear causal effects when pleiotropic proportion equals 50%. Overall, all four methods show improved MSE and RV accuracy in comparison with the results in Fig. 5. The comparison among the four methods shows a similar pattern to the scenario with a pleiotropic proportion of 100%. In the linear scenario, GAN-IV shows comparable performance to MR-LINK and DeLIVR, but all three methods outperform TSLS. In the nonlinear scenario, our proposed method presents more evident superiority among all the methods in both MSE and RV correlation, which validates the robustness of GAN-IV across different levels of pleiotropic proportions.

## Real data application

We apply the proposed framework to the ROSMAP dataset, which involves two prospective aging studies, the Religious Order Study (ROS) and the Memory and Aging Project (MAP) [22], to study the causal effect of genes on the development of Alzheimer’s disease (AD). We obtained matched genotype and gene expression data in the ROSMAP cohort. Specifically, the genotype data were obtained using whole-genome sequencing technology, and the gene expression profiles were obtained using microarray technology. Both are available via Synapse [23]. In this analysis, we focus on four key AD causal genes, which include early-onset AD causal genes (i.e. PSEN1, PSEN2, and APP) and late-onset AD (i.e. APOE). For each gene, variants within $\pm $500 kb of the gene coding region were extracted using bcftools v1.13 [24]. Genotypes were converted to a dosage format (0 = homozygous reference, 1 = heterozygous, 2 = homozygous alternate). Quality control was applied to retain only biallelic SNPs and to remove indels or sites with missing alternate alleles. Gene-level expression values were averaged across probes when multiple probes were mapped to the same gene and were normalized.

The phenotype of interest is the CERAD score, which originally was an ordinal variable summarized using the cognition test score. We transformed this score into a binary outcome using the recommended table provided by the institution [25]. The candidate genes and the number of variants in each gene are as follows: APOE ($14\,682$), APP ($13\,364$), PSEN1 ($13\,489$), PSEN2 ($12\,641$), with a sample size of 415 after data matching. The data preprocessing steps included removing SNPs with MAF <0.05 for all three genes, adjusting for covariates including sex, education level, race, and age. To amplify the genetic association, we regressed the transformed CERAD binary variable on covariates, then used the standardized residuals as the adjusted CERAD scores for model fitting. LD pruning was applied to all genes to remove SNPs with LD above 0.9 and leave one SNP to represent the information of window size 50.

For the IV selection, we adapted the procedure in He *et al*. [10]. Specifically, we fitted a linear regression of gene expression on the available SNPs and applied the backward selection with AIC to choose an optimal model and select the significant predictive SNPs. We also calculated the F statistics of the final model. Among all three genes, we identified PSEN2 with three IVs, APOE with three IVs, and APP with one IV, all with F statistics larger than 10. There were no significant SNPs selected by the procedure for PSEN1, hence no further results were reported for PSEN1. We applied TSLS, DeLIVR, and GAN-IV to the three genes with the selected IVs, normalized gene expression, and phenotypes. Note that MR-LINK is not applicable in this application as it requires additional summary statistics. To address the potential pleiotropic confounding, we identified an additional tag-SNP set and added them to the second stage estimation in the three methods. The number of variants in the tag-SNP set of three genes vary based on the LD range used to select tag-SNPs. The details of tag-SNP set are described in the appendix.

**Figure 7:**
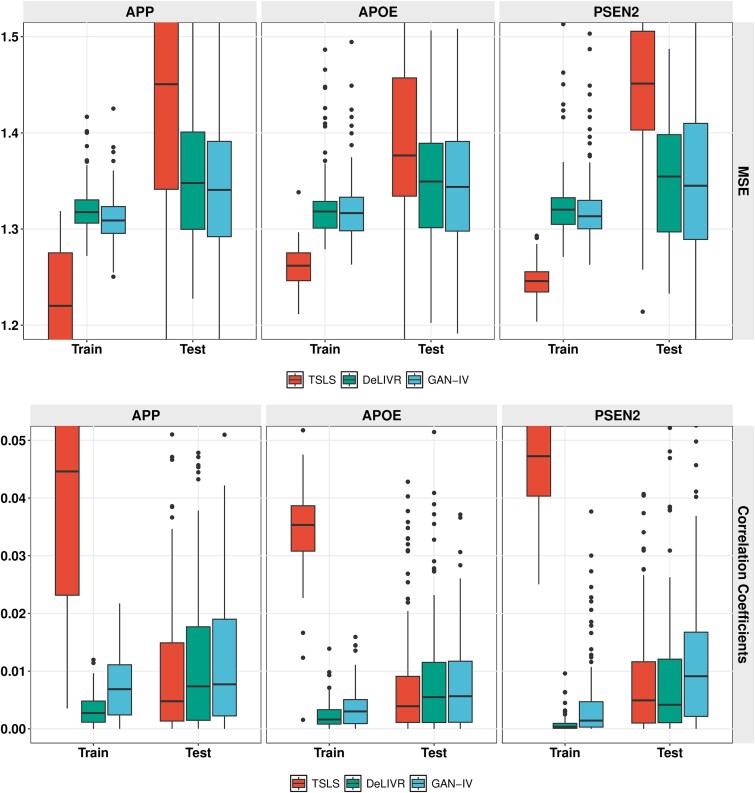
Causal effect estimation performance of the PSEN2, APOE, APP genes in the CERAD score.

To avoid chance findings caused by data splitting, we randomly split the dataset and repeated the experiments 100 times. For each run, the dataset was randomly divided into a training set and a testing set in a ratio of 4:1. MSE and RV correlation for the three methods are summarized in Fig. 7. GAN-IV achieves the highest estimation accuracy in both MSE and RV correlation among the three methods for all three genes. Despite similar deep learning structures in both GAN-IV and DeLIVR in the second stage, the superior performance of GAN-IV over DeLIVR may indicate that the GAN efficiently captured the exposure distribution in the first stage and hence improved the overall model accuracy. The computational cost of the three methods applied to each gene is included in the appendix.

**Figure 8:**
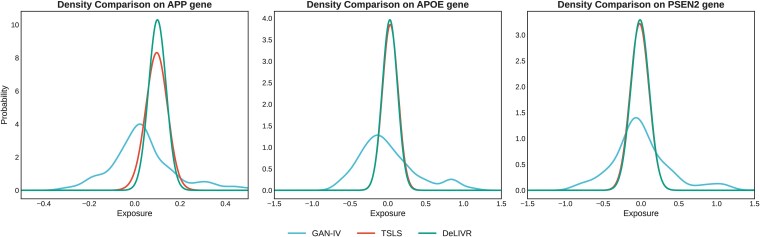
Exposure density estimation for the three genes conditioned on the sample median of IVs using GAN, TSLS, and DeLIVR.

We also conducted the exposure density estimation comparison estimated by GAN, TSLS, and DeLIVR, conditioning on the sample median of the selected IVs. We averaged the first stage exposure outputs $\hat g$ from 100 experiments of TSLS, DeLIVR and our proposed GAN-IV. For a fair comparison and informative illustration, we computed the KDEs for all three methods with a Gaussian kernel and a bandwidth selected by Scott’s rule of thumb. We evaluated the density estimators for all three methods at 1000 equally spaced points spanning the observed range of the true gene expression using densities estimated via KDE shown as in Fig. 8 showing the gene expression density of PSEN1, APOE, and APP. From the figure, we observed that both TSLS and DeLIVR reflect the normality assumption in their density estimates, whereas our GAN-based method learns the distribution directly from the data and captures the heavier tails of the exposure distribution across all three gene applications.

**Figure 9:**
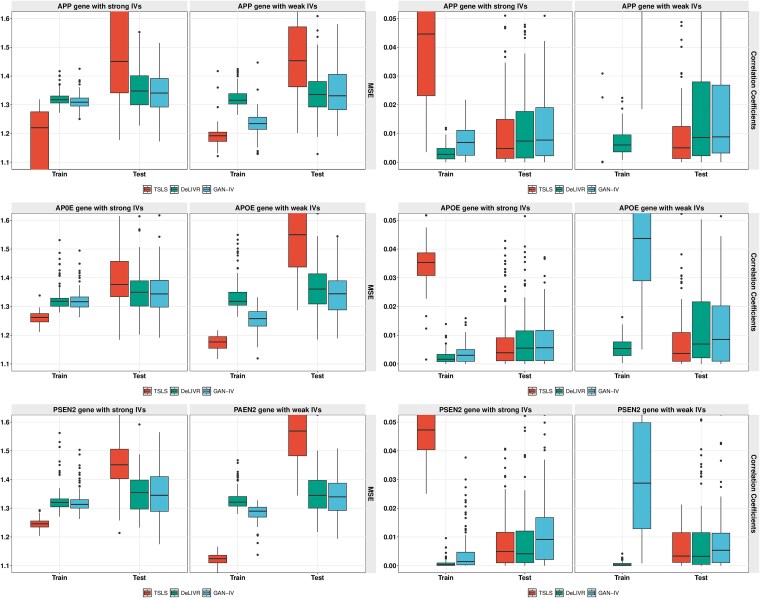
Causal effect estimation performance comparison of the PSEN2, APOE, APP genes in the CERAD score with strong and weak IVs.

To further assess the robustness of both stages of the proposed GAN-IV, we conducted two sensitivity analyses using the ROSMAP dataset: a weak IV scenario and a partial sample scenario. The weak IV scenario refers to IVs with moderate F-statistics ranging from 8 to 10, other data generating functions remain the same as in the previous analysis. The number of selected IVs is described in detail in the appendix. The comparison in terms of MSE and RV correlation between the strong IV scenario and the weak IV scenario across three genes is summarized in Fig. 9. By comparing two subpanels across three genes, it is observed that with weaker IVs, TSLS is prone to a higher level of overfitting. Although GAN-IV and DeLIVR also suffer from overfitting due to the violation of the strong IV assumption, GAN-IV outperforms TSLS and DeLIVR in all three genes. This is likely attributed to the ability of the conditional GAN model used in the first stage to capture signal from noise.

**Figure 10:**
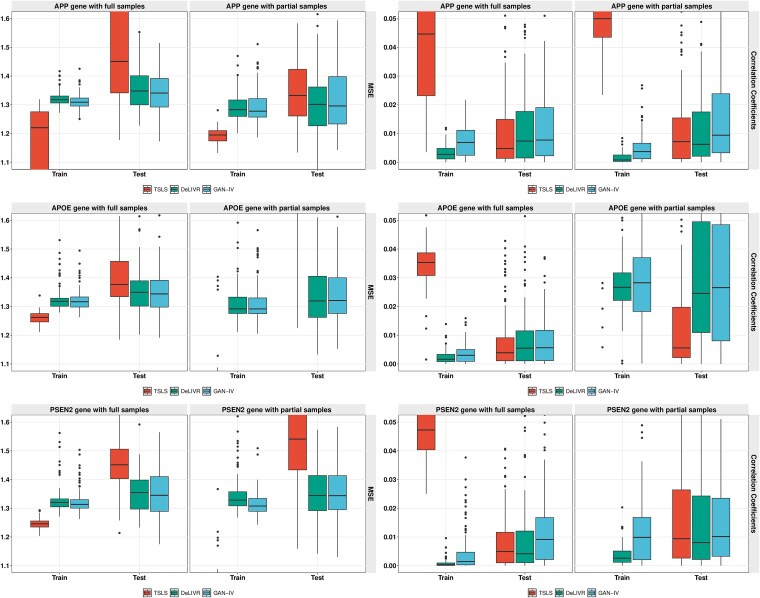
Causal effect estimation performance comparison of the PSEN2, APOE, APP genes in the CERAD score with full and partial samples.

Another scenario we considered as part of the sensitivity analysis is the evaluation of all three methods using a smaller sample size. We randomly selected 300 samples out of the ROSMAP data for each gene; MSE and RV correlation of all three methods are shown in Fig. 10. For GAN-IV and DeLIVR, which use DNN-based models, fewer samples usually lead to worse model fitting. This phenomenon is evidenced by the larger variance observed in the partial sample subpanels across all genes. In some cases, the MSE and RV correlation distributions also become skewed, where the median remains lower despite the higher placement of the box. While GAN-IV is more sensitive to sample size due to the usage of deep learning models in both stages, it still achieves comparable or better performance across all three genes.

Overall, in the sensitivity analysis, we validate the robustness of our model against the violation of an important assumption in causal inference and the limitation of sample size. It should be pointed out that in both scenarios, the tuning process for hyper-parameters will require more computational resources and iterations.

## Discussion

To summarize, we develop a two-stage nonparametric estimation for the causal effect in omics data, which uses SNPs as genetic IVs and gene expression as the exposure. In this research, we focus on several common problems faced by the causal analysis of omics data, the unknown distributions of omics data, LD and pleiotropic effects, and nonlinear exposure effects. To address these problems, we propose a nonparametric framework, GAN-IV, that provides robustness against omics data distribution assumptions, the ability to capture nonlinear causal effects, and consistency even in the presence of the IV condition violation (e.g. in the presence of pleiotropy). We extensively investigated both the exposure distribution estimation in the first stage and the modeling performance in the second stage. Compared with DeepIV, our approach avoids the inner-loop Monte Carlo step and ill-posed inversion of a conditional density. Compared with DeLIVR, we do not require an exponential-family or completeness condition on the exposure distribution. In terms of MR-LINK, which corrects for both LD effect and pleiotropy, our proposed method relieves the linear structure of the first-stage estimator and provides a more powerful model by accommodating extra tag-SNP information. Throughout the simulation studies and real data analysis, we elaborate on the above advantages by comparing the proposed framework with existing methods under different conditional densities, different exposure–outcome relationships, the presence of the LD effect and pleiotropy, and under different evaluation criteria. As demonstrated by both simulations and real data analysis, the proposed GAN-IV method achieves superior or comparable performance compared with the competing methods.

One possible limitation of the proposed approach lies in a failure mode of GAN called mode collapse. It refers to the situation in which a trained generator only outputs several fixed values regardless of the input. Although we do not observe this phenomenon in our model, for future applications, we can further improve the performance of GANs. Plenty of research has been conducted to improve the stability of the discriminator on the GAN-based models. Kurach *et al*. [26] conducted an analysis of several competing regularization and normalization methods on the GAN model. In our case, two possible extensions can be considered based on the conclusion in Kurach *et al*. [26]. When the computation resources are limited, we can add non-saturating GAN loss as proposed in Goodfellow *et al*. [13] and spectral normalization to the loss function. With enough resources, we can further add gradient penalty regularization or even switch the vanilla conditional GAN to a conditional version of Wasserstein GAN with gradient penalty, which is shown to provide better density estimation [27].

Another limitation of the proposed GAN-IV relates to the hyperparameter selection and the sample size requirement. Like most deep learning approaches, both the GAN in the first stage and the DFNN in the second stage of the proposed GAN-IV involve the choice of network architectures, the tuning of learning rates and regularization parameters, which typically require large sample sizes. Moreover, the complex modeling often requires a high computational cost. To mitigate these limitations, implementation details of the GAN-IV network structure and hyperparameter selection are provided in the appendix for practical guidance. Our experience in simulation and real data analysis shows that the cross-validation process is an effective tool for hyperparameter selection. The sensitivity analysis in the real data analysis indicates our proposed method achieves higher accuracy even with a limited sample size of 300. The computational costs provided in the appendix show relatively affordable computational time of GAN-IV compared with existing methods, given its improved performance.

**Key Points**

- The two-stage GAN-based instrument variable method (GAN-IV) utilizes conditional generative adversarial networks to estimate the conditional distribution of gene expression given IVs in the first stage and the functional neural networks to model the causal effects between gene expression and the phenotype.
- The GAN-IV method is free of distribution assumptions of the exposure variables and is powerful to capture nonlinear causal effects compared with other two-stage methods such as the two-stage least squares method and MR-LINK.
- In the second stage of GAN-IV, the functional neural network structure is flexible to handle complex omics data (e.g. tag-SNPs) in the presence of pleiotropy and linkage disequilibrium.

## Supplementary Material

supplement_bbag071

## Data Availability

The source code for the simulation and real data application is available at https://github.com/DianaYuanZhou/GAN-IV.git. The ROSMAP dataset used in this article is available at https://www.synapse.org/Synapse:syn10901595.

## References

[1] Sanderson E, Glymour MM, Holmes MV et al. Mendelian randomization. *Nat Rev Methods Primers* 2022;2:6. 10.1038/s43586-021-00092-5 37325194 PMC7614635

[2] Bowden J, Davey Smith G, Burgess S. Mendelian randomization with invalid instruments: effect estimation and bias detection through Egger regression. *Int J Epidemiol* 2015;44:512–25. 10.1093/ije/dyv080 26050253 PMC4469799

[3] Verbanck M, Chen C-Y, Neale B et al. Detection of widespread horizontal pleiotropy in causal relationships inferred from Mendelian randomization between complex traits and diseases. *Nat Genet* 2018;50:693–8. 10.1038/s41588-018-0099-7 29686387 PMC6083837

[4] Barfield R, Feng H, Gusev A et al. Transcriptome-wide association studies accounting for colocalization using Egger regression. *Genet Epidemiol* 2018;42:418–33. 10.1002/gepi.22131 29808603 PMC6342197

[5] van Der Graaf A et al. Mendelian randomization while jointly modeling cis genetics identifies causal relationships between gene expression and lipids. *Nat Commun* 2020;11:4930. 10.1038/s41467-020-18716-x 33004804 PMC7530717

[6] Newey WK . Nonparametric instrumental variables estimation. *Am Econ Rev* 2013;103:550–6. 10.1257/aer.103.3.550

[7] Hall P, Horowitz JL. Nonparametric methods for inference in the presence of instrumental variables. *Ann Stat* 2005;33:2904–29. 10.1214/009053605000000714

[8] Staley JR, Burgess S. Semiparametric methods for estimation of a nonlinear exposure-outcome relationship using instrumental variables with application to Mendelian randomization. *Genet Epidemiol* 2017;41:341–52. 10.1002/gepi.22041 28317167 PMC5400068

[9] Hartford J, Lewis G, Leyton-Brown K et al. Deep IV: a flexible approach for counterfactual prediction. In: Doina Precup, Yee Whye Teh (eds.), International Conference on Machine Learning, pp. 1414–23. Sydney, Australia: PMLR, 2017.

[10] He R, Liu M, Lin Z et al. DeLIVR: a deep learning approach to IV regression for testing nonlinear causal effects in transcriptome-wide association studies. *Biostatistics* 2024;25:468–85. 10.1093/biostatistics/kxac051 36610078 PMC11017120

[11] Abbasnejad ME, Shi Q, Hengel A v d et al. A generative adversarial density estimator. In: Proceedings of the IEEE/CVF Conference on Computer Vision and Pattern Recognition, Long Beach, CA, USA, pp. 10782–91, 2019.

[12] Zhou Y, Geng P, Zhang S et al. Multimodal functional deep learning for multiomics data. *Brief Bioinform* 2024;25:bbae448. 10.1093/bib/bbae448 39285512 PMC11405129

[13] Goodfellow IJ et al. Generative adversarial nets. *Advances in neural information processing systems* Ghahramani Z, Welling M, Cortes C, Lawrence N, Weinberger KQ (eds.). Montreal, Quebec, Canada, 2014;27.

[14] Mirza M, Osindero S. Conditional generative adversarial nets. *arXiv* 2014; Preprint arXiv:1411.1784

[15] Mescheder L, Geiger A, Nowozin S. Which training methods for GANs do actually converge? In: Dy J, Krause A (eds.), *Proceedings of the 35th International Conference on Machine Learning, Vol. 80 of Proceedings of Machine Learning Research*, pp. 3481–90. Stockholmsmässan, Stockholm Sweden: PMLR, 2018.

[16] Kingma D, Adam P. A method for stochastic optimization. *arXiv* 2014; Preprint arXiv:1412.6980

[17] Nguyen X, Wainwright MJ, Jordan MI. Estimating divergence functionals and the likelihood ratio by convex risk minimization. *IEEE Trans Inf Theory* 2010;56:5847–61. 10.1109/TIT.2010.2068870

[18] Gretton A, Borgwardt KM, Rasch MJ et al. A kernel two-sample test. *J Mach Learn Res* 2012;13:723–73.

[19] Zhang S, Zhou Y, Geng P et al. Functional neural networks for high-dimensional genetic data analysis. *IEEE/ACM Trans Comput Biol Bioinform* 2024;21:383–93. 10.1109/TCBB.2024.3364614 38507390 PMC11301578

[20] Consortium, G. P et al. A global reference for human genetic variation. *Nature* 2015;526:68. 26432245 10.1038/nature15393 PMC4750478

[21] Su Z, Marchini J, Donnelly P. HAPGEN2: simulation of multiple disease SNPs. *Bioinformatics* 2011;27:2304–5. 10.1093/bioinformatics/btr341 21653516 PMC3150040

[22] Bennett DA, Buchman AS, Boyle PA et al. Religious orders study and rush memory and aging project. *J Alzheimer’s Dis* 2018;64:S161–89. 10.3233/JAD-179939 29865057 PMC6380522

[23] Sage Bionetworks . i. Genomic Variants (Whole Genome Sequencing)—synapse.org. https://www.synapse.org/Synapse:syn10901595.

[24] Li H . A statistical framework for SNP calling, mutation discovery, association mapping and population genetical parameter estimation from sequencing data. *Bioinformatics* 2011;27:2987–93. 10.1093/bioinformatics/btr509 21903627 PMC3198575

[25] Mirra SS, Heyman A, McKeel D et al. The consortium to establish a registry for Alzheimer’s disease (CERAD) part II. Standardization of the neuropathologic assessment of Alzheimer’s disease. *Neurology* 1991;41:479–9. 10.1212/WNL.41.4.479 2011243

[26] Kurach K, Lucic M, Zhai X et al. A large-scale study on regularization and normalization in GANs. In: Kamalika Chaudhuri, Ruslan Salakhutdinov (eds.), International conference on machine learning, pp. 3581–90. Long Beach, CA, USA: PMLR, 2019.

[27] Gulrajani I, Ahmed F, Arjovsky M et al. Improved training of Wasserstein GANs. Guyon I, Von Luxburg U, Bengio S, Wallach H, Fergus R, Vishwanathan S, Garnett R (eds.), *Advances in neural information processing systems*. Long Beach, CA, USA, 2017;30.

