# Supplementary material for: A two-stage GAN-based instrumental variable method for causal analysis of omics data
Source: Brief Bioinform. 2026 Feb 23;27(1):bbag071. doi: 10.1093/bib/bbag071 (PMC12928005; doi:10.1093/bib/bbag071)
Supplement: supplement_bbag071 [file supplement_bbag071.pdf]

Appendix

Simulation settings and model implementation

In both simulations 1 and 2, the IV SNPs are generated as

$$Z_j \sim Multinomial(n = 1000, prob = (0.64, 0.32, 0.04)), \quad j = 1, .., p \tag{1}$$

Simulation 1

The density function of exposure g is simulated as: $G = 0.5Z + \varepsilon_G, \varepsilon_G \sim p_{\varepsilon_G}$

The choices of p_{ε_G} including:

1. Normal: $p_{\varepsilon_G}(x) = \mathcal{N}(0, 0.5)$
2. Laplace: $p_{\varepsilon_G}(x) = Laplace(2, 2)$
3. Gamma distribution: $p_{\varepsilon_G}(x) = Gamma(2, 0.5)$
4. Mixture Normal: $p_{\varepsilon_G}(x) = 0.4\mathcal{N}(0.5, 0.2) + 0.6\mathcal{N}(4, 0.3)$

We assume that Y follows a linear relationship with G and generate Y as a scalar following the relationship below.

$$Y_0 = 1 + \beta' G + U, \quad Y = Y_0 + \varepsilon_Y, \quad \varepsilon_Y \sim N(0, c_1 var(Y_0)),$$

where the causal effect size $\beta_j = 0.8$ for all j. U is the unobserved confounding factor, and c_1 is a constant controlling the signal-noise ratio. We assume U is correlated with ε_G by $U = R'\varepsilon_G$, where $R = (0.8, 0.8, 0.8, 0.8, 0.8)$.

Table 1. Implementation details and hyperparameter settings for conditional GAN and linear density estimator in Simulation 1

Stage	Hyperparameter	GAN-IV (Proposed)	Linear density estimator
Stage 1: Exposure Distribution Estimation			
	Model Architecture	Conditional GAN (Generator: 3 hidden layers with [50,30,30] hidden nodes; Discriminator: 3 hidden layers with [50,30,10])	Linear Regression
	Noise distribution	$s_{i,1}, ...s_{i,50} \sim Unif(-5, 5)$	
	Activation Function	Generator: Tanh (hidden), Linear (Output); Discriminator: ELU (hidden), Sigmoid (output)	N/A
	Loss Function	Non-saturating generator loss; BCE discriminator loss	Least Squares
	Optimization	Adam Optimizer with learning rate 0.0001	Closed-form solution
	Training Duration	50,000 Epochs (Fixed)	N/A

Simulation 2

The exposure variable and outcome are simulated as:

$$G = 0.5Z + \varepsilon_G, \quad \varepsilon_G \sim \mathcal{N}(0.5 + Z^2, 0.3 + 0.5 * Z^2 + 1e^{-8}), \quad Y_0 = f(G) + U, \quad Y = Y_0 + \varepsilon_Y$$

1. Cosine function: $f(G) = 0.5cos(\frac{\pi}{2}(0.6g-1)), U = 0.35\varepsilon_G$
2. Polynomial function: $f(G) = 0.3(0.5(g-(1-mean(g))/std(g))^3) + 2|g|^{1/3}, U = 0.25\varepsilon_G$

The two-way threshold and three-way threshold functions are defined as:

$$G = 0.5Z + \varepsilon_G, \quad \varepsilon_G \sim \mathcal{N}(0,1), \quad Y_0 = f(G) + U, \quad Y = Y_0 + \varepsilon_Y, \quad \varepsilon_Y \sim N(0, c_2 Var(Y_0))$$

1. Linear function with two-way threshold interaction: $f(G) = 0.3g + 0.8\sum_{j\neq k} g_j g_k I(|g_j g_k| > 2), U = 0.25\varepsilon_G$
2. Linear function with three-way threshold interaction: $f(G) = 0.3g + 0.6\sum_{j\neq k\neq l} g_j g_k g_l I(|g_j g_k g_l| > 2), U = 0.25\varepsilon_G$

Table 2. Implementation details and hyperparameter settings for GAN-IV and DeLIVR in simulation 2

Stage	Hyperparameter	GAN-IV (Proposed)	DeLIVR
Stage 1: Exposure Distribution Estimation			
	Model Architecture	Conditional GAN (Generator:1 hidden layers with [50] hidden nodes; Discriminator: 1 hidden layers with [50] hidden nodes)	Linear Regression
	Noise distribution	$s_{i,1},...s_{i,50} \sim Unif(-5,5)$	
	Activation Function	Generator: ELU (hidden), Linear (Output); Discriminator: ELU (hidden), Sigmoid (output)	N/A
	Loss Function	Non-saturating generator loss; BCE discriminator loss with R_1 penalty	Least Squares
	Optimization	Adam Optimizer with learning rate 0.0001	Closed-form solution
	Training Duration	50,000 Epochs (Fixed)	N/A
Stage 2: Causal Effect Estimation			
	Model Architecture	Deep Functional Neural Network with 2 hidden layers [20,10]	Deep Neural Network with 5 hidden layers [32,16,8,8,8]
	Basis Expansion	B-Spline (n_{basis} = 10, order 3)	N/A
	Activation Function	ReLU (hidden), Linear (output)	Sigmoid
	Loss Function	Mean Squared Error (MSE)	Mean Squared Error (MSE)
	Regularization	L_2 Regularization selected by 3-fold cross validation	N/A
	Optimization	Adam Optimizer with learning rate selected by a 3-fold cross validation	Adam Optimizer with decay learning rate starting at 0.0001
	Stopping Criteria	Early stopping (Patience = 20); Max Epochs = 10,000	Early stopping (Patience = 20); Max Epochs = 5,000

Simulation 3

We simulated the two exposure sets as follows.

$$G_E = \sum_{j=1}^{10} Z_{E,j}\beta_{E,j} + C_E + \varepsilon_E, \quad G_U = \sum_{j=1}^{10} Z_{U,j}\beta_{U,j} + C_U + \varepsilon_U, \tag{2}$$

where $\beta_{E,j}, \beta_{U,j} \sim Unif(-0.5, 0.5)$. The confounder factors C_E, C_U and the noises ε_E, ε_U all independently follow $N(0, 0.09)$. The phenotype y is generated from both linear and nonlinear causal effect models as follows.

$$\text{linear}: \quad Y = G_E + 0.4G_U + \varepsilon_Y,$$
$$\text{nonlinear}: \quad Y = G_E + (G_E - \bar{G}_E)^2 + sin(2\pi(G_E - \bar{G}_E)) + 0.4G_U + \varepsilon_Y, \quad \varepsilon_Y \sim N(0, 0.09).$$

Table 3. Implementation details and hyperparameter settings for GAN-IV and DeLIVR in simulation 3

Stage	Hyperparameter	GAN-IV (Proposed)	DeLIVR
Stage 1: Exposure Distribution Estimation			
	Model Architecture	Conditional GAN (Generator: 2 hidden layers with [50, 25] hidden nodes; Discriminator: 1 hidden layers with [5] hidden nodes)	Linear Regression
	Noise distribution	$s_{i,1}, ...s_{i,50} \sim Unif(-5, 5)$	
	Activation Function	Generator: ELU (hidden), Linear (Output); Discriminator: ELU (hidden), Sigmoid (output)	N/A
	Loss Function	Non-saturating generator loss; BCE discriminator loss	Least Squares
	Optimization	Adam Optimizer with 0.0001 (G) and 0.001 (D) learning rate	Closed-form solution
	Training Duration	50,000 Epochs (Fixed)	N/A
Stage 2: Causal Effect Estimation			
	Model Architecture	Multi-modal Functional Neural Network (MFNN) (Hidden nodes [[3],[10]],[10])	Deep Neural Network with 3 hidden layers (Hidden nodes [4,2,2])
	Basis Expansion	B-Spline (n_{basis} = 20, order 3)	N/A
	Activation Function	ReLU (hidden), Linear (output)	Sigmoid
	Loss Function	Mean Squared Error (MSE)	Mean Squared Error (MSE)
	Regularization	L_2 Regularization selected by 3-fold cross validation	N/A
	Optimization	Adam Optimizer with learning rate selected by 3-fold cross validation	Adam Optimizer with decayed learning rate starting at 0.001
	Stopping Criteria	Early stopping (Patience = 10); Max Epochs = 10,000	Early stopping (Patience = 10); Max Epochs =10,000

Data preparation in real data analysis

Table 4. Summary of F-statistics and IV selection numbers for different genes.

Gene	F stat	IV filtered	Selected IV number after removing overlapped IVs
APP	10.19	1	1
	8.454	2	1
APOE	10.59	1	1
	8.733	6	4
PSEN2	11.92	3	3
	9.209	3	2

Among PSEN2, APOE, APP genes used in strong IV analysis, the LD range for selecting tag-SNPs and number of variant in tag-SNP set are:

1. APP: LD between [0.3, 0.99], tag-SNP set has 7 variants
2. APOE: LD between [0.5, 0.99], tag-SNP set has 15 variants
3. PSEN2: LD between [0.5, 0.99], tag-SNP set has 21 variants

In weak IV scenario, the LD range for selecting tag-SNPs and number of variant in tag-SNP set are:

1. APP: LD between [0.2, 0.99], tag-SNP set has 26 variants
2. APOE: LD between [0.3, 0.99], tag-SNP set has 37 variants
3. PSEN2: LD between [0.3, 0.99], tag-SNP set has 51 variants

Table 5. Implementation details and hyperparameter settings for GAN-IV and DeLIVR in Real Data Analysis

Stage	Hyperparameter	GAN-IV (Proposed)	DeLIVR
Stage 1: Exposure Distribution Estimation			
	Model Architecture	Conditional GAN (Generator: 4 hidden layers with [50,25,12,6] hidden nodes; Discriminator: 3 hidden layers with [10,10,5] hidden nodes)	Linear Regression
	Noise distribution	$s_{i,1},...s_{i,50} \sim Unif(-5,5)$	
	Activation Function	Generator: ELU (hidden), Linear (Output); Discriminator: ELU (hidden), Sigmoid (output)	N/A
	Loss Function	Non-saturating generator loss; BCE discriminator loss with R_1 penalty	Least Squares
	Optimization	Adam Optimizer with 0.0001 (D) and 0.001 (G) learning rate	Closed-form solution
	Training Duration	50,000 Epochs (Fixed)	N/A
Stage 2: Causal Effect Estimation			
	Model Architecture	Multi-modal Functional Neural Network (MFNN) (Hidden nodes [[3],[5]],[5])	Neural Network with 2 hidden layers (Hidden nodes [10,5])
	Basis Expansion	B-Spline (n_{basis} = 10, order 3)	N/A
	Activation Function	ReLU (hidden), Linear (output)	Sigmoid
	Loss Function	Mean Squared Error (MSE)	Mean Squared Error (MSE)
	Regularization	L_2 Regularization selected by 3-fold cross validation	N/A
	Optimization	Adam Optimizer with learning rate selected by a 3-fold cross validation	Adam Optimizer with a decayed learning rate starting at 0.0001
	Stopping Criteria	Early stopping (Patience = 3); Max Epochs = 10,000	Early stopping (Patience = 50); Max Epochs = 20,000

Computational cost comparison in real data analysis

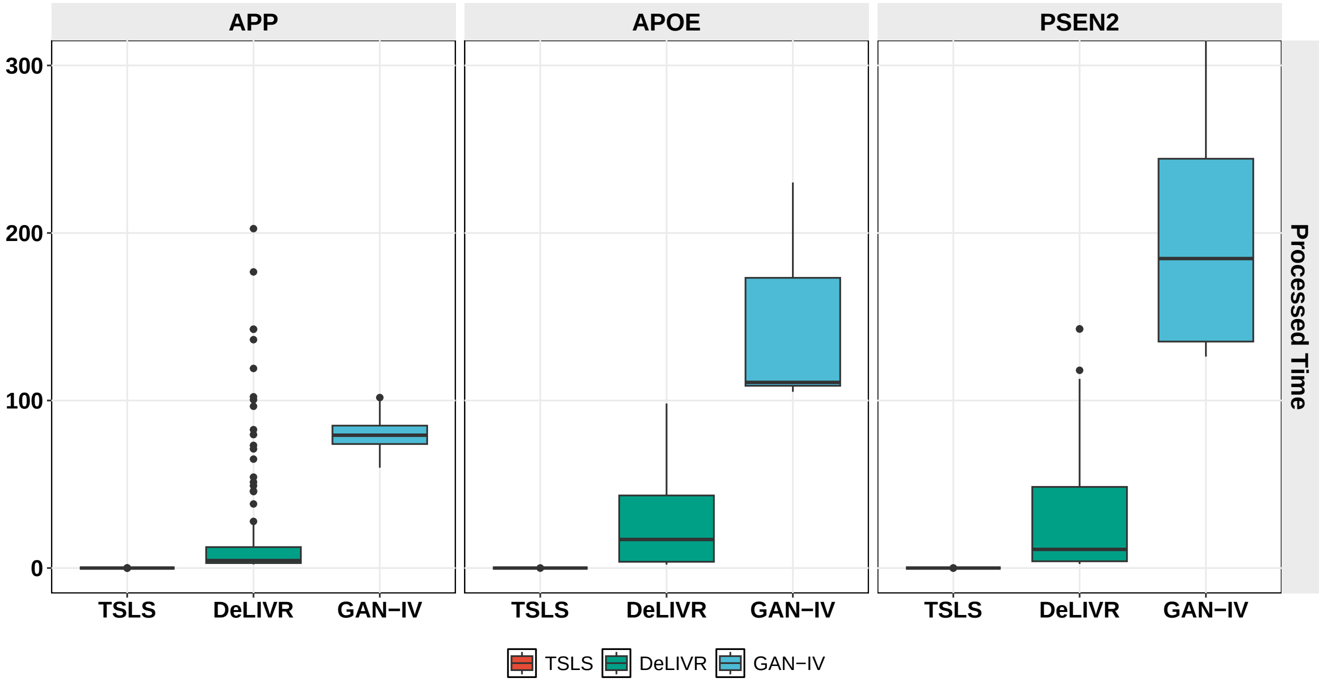

Figure 1. Computational cost of TSLS, DeLIVR and GAN-IV applied to gene APP, APOE and PSEN2.
